# 1100. A Prospective Evaluation of Neurotoxicity Among Patients Receiving Dose-Optimized Cefepime or Meropenem With Concomitant Therapeutic Drug Monitoring

**DOI:** 10.1093/ofid/ofab466.1294

**Published:** 2021-12-04

**Authors:** Brandon Smith, Ellen G Kline, Lori Shutter, Joanna Fong-Isariyawongse, Alexandra Urban, Holt Murray, Karin Byers, Ryan K Shields

**Affiliations:** 1 University of Pittsburgh Medical Center, Pittsburgh, Pennsylvania; 2 University of Pittsburgh, School of Medicine, Pittsburgh, PA; 3 University of Pittsburgh, Pittsburgh, Pennsylvania

## Abstract

**Background:**

Cefepime (FEP) induced neurotoxicity (NT) may have serious implications for patients (pts). Retrospective studies have employed variable definitions of NT, finding renal impairment and FEP trough concentrations (Cmin) > 20 mg/L as risk factors. Prospective studies comparing antibiotics have not been performed.

**Methods:**

We conducted a prospective study of pts receiving FEP or meropenem (MEM) with neurologic evaluation and therapeutic drug monitoring (TDM). A NT advisory board (NTAB) was established to develop standardized definitions of possible, probable and definitive NT (Fig 1). Cases of potential NT were adjudicated by the NTAB who were blinded to study treatment. FEP and MEM midpoint and Cmin concentrations were measured at steady-state by validated methods.

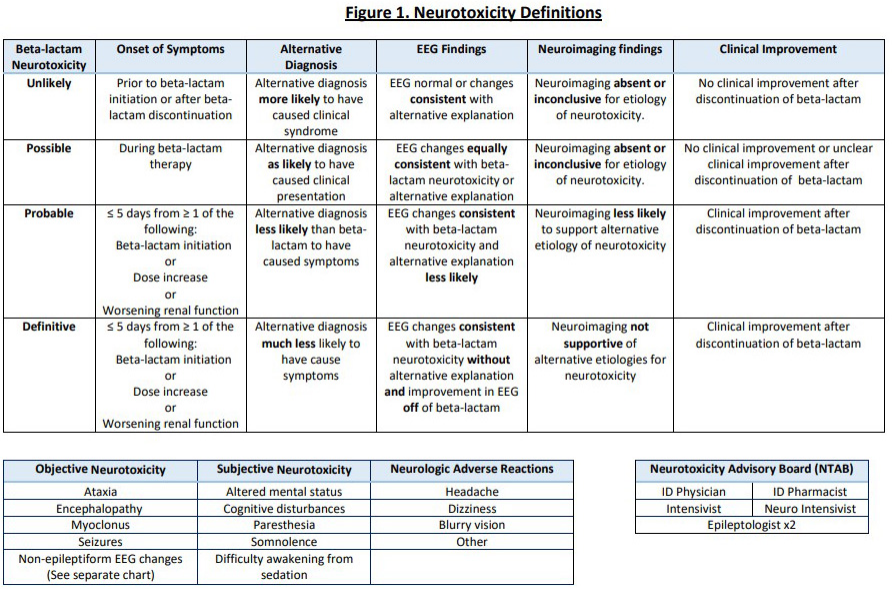

**Results:**

127 patients were included (70 FEP, 57 MEM). Demographics and treatment characteristics were similar between groups (Fig 2); 63% were in the ICU. FEP and MEM Cmin varied from 1.9 – 140.5 and 0.6 – 31.3 mg/L, respectively. Median FEP Cmin and total exposures (AUC) were 23.1 mg/L and 347.6 hr*mg/L, respectively. Corresponding MEM values were 5.9 mg/L and 124.8 hr*mg/L, respectively. Cmin values were inversely correlated with renal function for both FEP and MEM (*P*< 0.001). Rates of possible, probable, or definitive NT were 10% and 5% for FEP and MEM, respectively (*P=*0.51; Fig 3). 16% and 3% of pts with FEP Cmin > or < 20 mg/L had NT, respectively (*P=*0.11; Fig 4). Median MEM Cmin were 12.3 and 5.4 mg/L among pts with and without NT, respectively (*P=*0.09; Fig 4). Rates of NT did not vary by infusion length or dose. FEP and MEM exposures were similar between patients with (17%) or without (83%) microbiologic recurrence due to the same pathogen. FEP was discontinued in 4 pts due to NT; no pts stopped MEM due to NT.

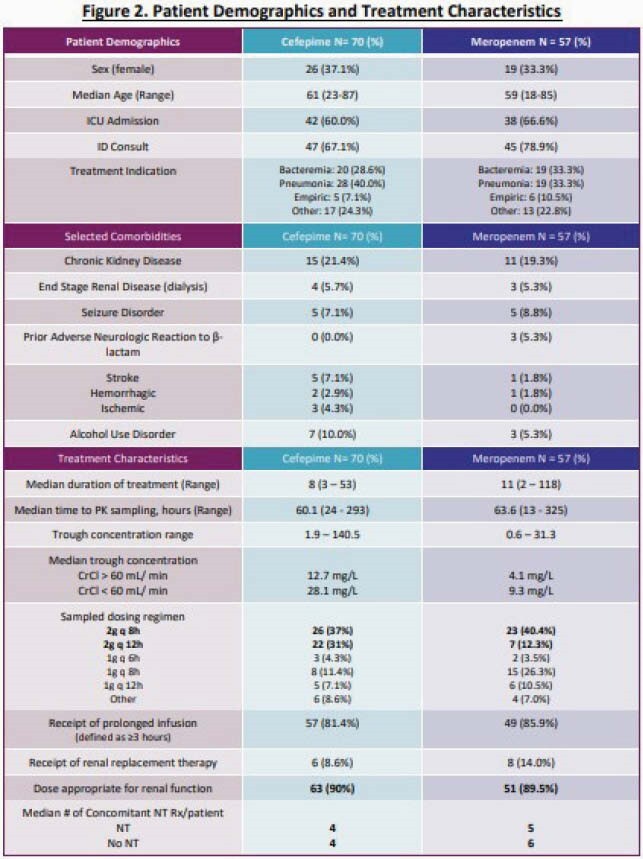

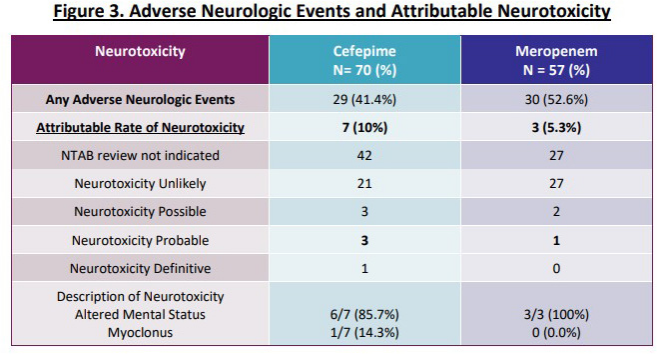

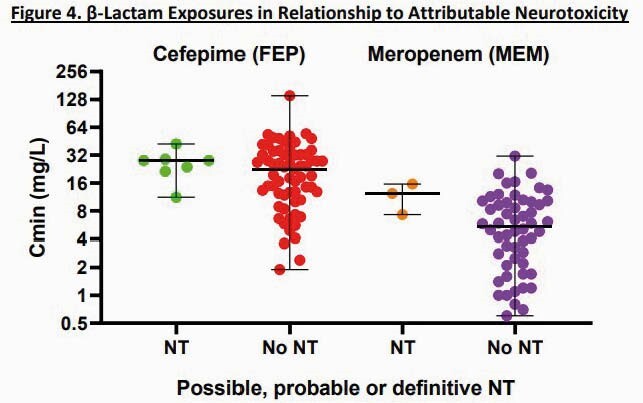

**Conclusion:**

Our study is the first to evaluate FEP NT prospectively and compare rates of NT to pts receiving MEM. We established criteria that were applied by a blinded NTAB. In doing so we found rates of NT to be lower than previously reported and not statistically different between FEP and MEM. Cmin values were highly variable and associated with numerically, but not statistically higher rates of NT for both agents. These findings serve as the basis for larger, multicenter studies and justify use of routine TDM to limit NT among high-risk pts.

**Disclosures:**

**Brandon Smith, MD, PharmD**, **Shionogi** (Consultant, Advisor or Review Panel member) **Alexandra Urban, MD**, **Neuropace** (Consultant) **Ryan K. Shields, PharmD, MS**, **Shionogi** (Consultant, Research Grant or Support)

